# A CACNA1D mutation in a patient with persistent hyperinsulinaemic hypoglycaemia, heart defects, and severe hypotonia

**DOI:** 10.1111/pedi.12512

**Published:** 2017-03-20

**Authors:** SE Flanagan, F Vairo, MB Johnson, R Caswell, TW Laver, H Lango Allen, K Hussain, S Ellard

**Affiliations:** ^1^ Institute of Biomedical and Clinical ScienceUniversity of Exeter Medical SchoolExeterUK; ^2^ Medical Genetics ServiceHospital de Clínicas de Porto AlegrePorto AlegreRSBrazil; ^3^ Developmental Endocrinology Research Group, Clinical and Molecular Genetics UnitUCL Institute of Child Health and Great Ormond Street HospitalLondonUK

**Keywords:** CACNA1D, calcium channel, exome sequencing, hyperinsulinism, hypoglycaemia

## Abstract

Congenital hyperinsulinaemic hypoglycaemia (HH) can occur in isolation or it may present as part of a wider syndrome. For approximately 40%‐50% of individuals with this condition, sequence analysis of the known HH genes identifies a causative mutation. Identifying the underlying genetic aetiology in the remaining cases is important as a genetic diagnosis will inform on recurrence risk, may guide medical management and will provide valuable insights into β‐cell physiology. We sequenced the exome of a child with persistent diazoxide‐responsive HH, mild aortic insufficiency, severe hypotonia, and developmental delay as well as the unaffected parents. This analysis identified a de novo mutation, p.G403D, in the proband's CACNA1D gene. CACNA1D encodes the main L‐type voltage‐gated calcium channel in the pancreatic β‐cell, a key component of the insulin secretion pathway. The p.G403D mutation had been reported previously as an activating mutation in an individual with primary hyper‐aldosteronism, neuromuscular abnormalities, and transient hypoglycaemia. Sequence analysis of the CACNA1D gene in 60 further cases with HH did not identify a pathogenic mutation. Identification of an activating CACNA1D mutation in a second patient with congenital HH confirms the aetiological role of CACNA1D mutations in this disorder. A genetic diagnosis is important as treatment with a calcium channel blocker may be an option for the medical management of this patient.

Hyperinsulinaemic hypoglycaemia (HH) is characterized by unregulated insulin secretion despite low blood glucose with prompt treatment essential in order to avoid seizures and permanent brain injury. Diazoxide is an effective treatment for the majority of cases with HH but for those who do not respond somatostatin analogues may sometimes be effective.^1^, ^2^

Persistent HH is most commonly a result of a monogenic aetiology with mutations in 9 genes described over the past 20 years.^2^ Each responsible gene was discovered either by linkage studies or via a candidate gene approach and collectively mutations in these genes are identified in 40%‐50% of cases with persistent HH.^3^ An absence of mutations in the remainder of patients suggests that novel etiological genes remain to be discovered.

In addition to the 9 recognized monogenic subtypes of HH, mutations in many genes have been reported where HH is a feature of a syndrome in some individuals.^4^ It therefore seems likely that gene discovery studies to search for novel HH genes will identify rare mutations in known multisystem disease‐causing genes where HH has not previously been reported or is a minor feature.

Whilst next‐generation sequencing (NGS) has revolutionized gene discovery studies outside of this condition, no novel disease genes have been identified in patients with persistent HH. There are likely to be several reasons for this; it may reflect the genetic heterogeneity of HH which makes replication studies to confirm pathogenicity of novel variants more difficult or that the HH in the patients previously screened is not monogenic. As patients with persistent early‐onset syndromic disease have the highest probability of having a monogenic aetiology these individuals represent good candidates for gene discovery studies.

## 1 METHODS

We undertook exome sequencing in an individual with persistent HH diagnosed at birth and her unaffected parents to search for a de novo mutation as previously described.^5^ Mutations in the 9 known HH genes had been excluded. The patient was diagnosed with HH shortly after birth when blood glucose was found to be 1.2 mmol/L (22 mg/dL) with corresponding insulin 21 μU/mL; she was treated with diazoxide(13 mg/kg/day). She was born large for gestational age with a birth weight greater than the 99th percentile, consistent with increased insulin secretion in utero. Bradycardia was detected on a prenatal echocardiogram at 27 weeks gestation with mild aortic insufficiency diagnosed at birth. Additional features included umbilical hernia, hypermetropia, severe axial hypotonia, and seizures (Table 1). At the age of 5 years, the patient no longer required diazoxide therapy and currently remains off all treatment for HH at the age of 9 years 4 months. The study was conducted in accordance with the Declaration of Helsinki with informed parental consent given on behalf of the child.

**Table 1:** Clinical characteristics of the proband with a de novo p.Gly403Asp *CACNA1D* mutation

Sex	Female
Birth weight (gestation)	4.5 kg (37 weeks)
Birth weight percentile	>99th
Current age	9 years 4 months
Hyperinsulinaemic hypoglycaemia	
Age at diagnosis	Birth
Insulin at diagnosis	21 μU/mL
Glucose at diagnosis	1.2 mmol/L (22 mg/dL)
Initial treatment (dose) and duration	Diazoxide (13 mg/kg/day) 5.5 years
Current treatment	None
Additional clinical features	
Heart defects	Prenatal bradycardia, mild aortic insufficiency
Neuromuscular defects	Severe axial hypotonia and limb spasticity, seizures
Other features	Umbilical Hernia, Hypermetropia. Poor weight gain
Aldosterone (normal range)	9.3 ng/dL (3.4‐27.3)

Target enrichment was carried out using the Agilent SureSelect All Exon v5 capture kit (Agilent, Santa Clara, California). 100‐bp paired‐end reads were sequenced on a HiSeq 2 500 (Illumina, San Diego, California). Analysis was carried out following the best practice guidelines for GATK from the Broad Institute (https://software.broadinstitute.org/gatk/best‐practices/). Sequence reads were aligned to the hg19/GRCh37 human reference genome with BWA‐MEM with the mean target coverage for the proband of 64 reads per base and 91% of targeted bases covered by at least 20 reads. Variants were called using GATK and annotated with Alamut (Rouen, France). Heterozygous variants were filtered by removing non‐coding and synonymous variants, variants in the Exome Aggregation Consortium or databases single nucleotide polymorphism (dbSNP) and those present in either parent. De novo mutations were confirmed by Sanger sequencing (details of primers are available on request).

The 49 coding exons and the intron/exon boundaries of *CACNA1D* (NM_000720.3) were sequenced using targeted NGS in 60 individuals with diazoxide‐treated HH diagnosed before 12 months in whom mutations in the 9 known genes had been excluded. In this cohort, the median age at diagnosis of HH was 3 weeks (range: 0‐12 months). Six patients had cardiac defects which included a heart murmur (n = 1), patent ductus arteriosus (n = 1), ventricular septal defect (n = 2), or hypertrophic cardiomyopathy (n = 2).

## 2 RESULTS AND DISCUSSION

**Figure 1:**
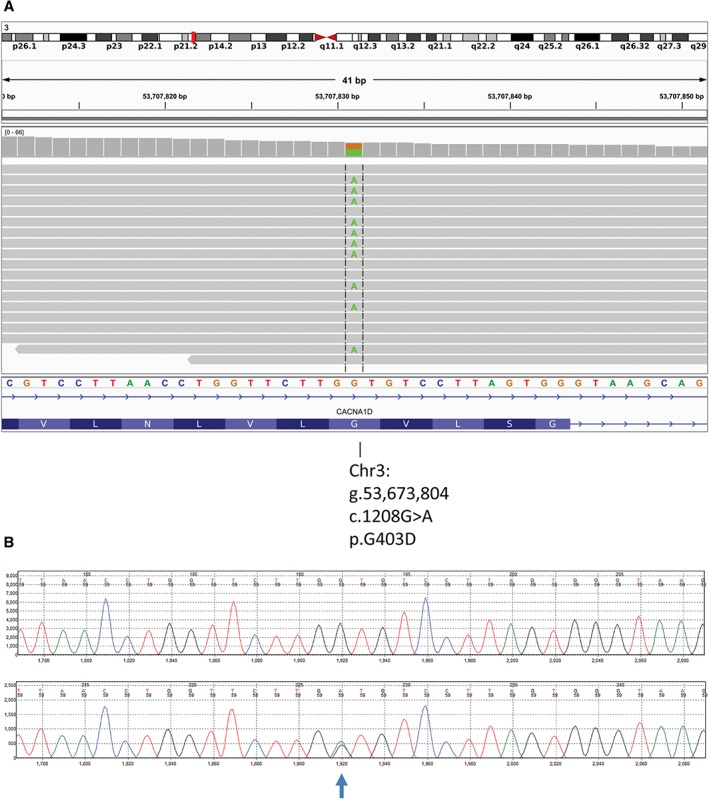
A, An integrative genomics viewer (IGV) screenshot showing the sequencing reads (grey bars) mapping to exon 8 of the CACNA1D gene located at genomic position g.53,673,804 on chromosome 3. The reference nucleotide sequence and the amino acid translation are provided under the sequencing reads. The heterozygous substitution of a guanine (G) to an adenine (A) at nucleotide position 1208 in 8 of the 18 sequencing reads is highlighted in green. B, The Sanger sequencing trace for the variant. The top trace is the reference while the bottom is the patient's sample. The heterozygous substitution of a guanine (G) to an adenine (A) is highlighted.

Exome sequencing of this family trio identified a single de novo missense substitution, p.Gly403Asp (c.1208G>A) in the *CACNA1D* gene (figure 1). Analysis of the sequence traces showed that the alleles were balanced, suggesting that this was more likely to be a germline mutation rather than a postzygotic event. In silico analysis predicted the variant was likely to be pathogenic and that the affected residue was highly conserved (Alamut). According to the recommendations of the American College of Medical Genetics and Genomics and the Association of Molecular pathology the evidence to support pathogenicity of this variant is strong.^6^

Targeted NGS of *CACNA1D* in 60 further patients did not identify a pathogenic variant. The absence of mutations may reflect the low proportion of individuals with cardiac disease in the cohort (6 of 60) or it may represent the low prevalence of *CACNA1D* mutations in individuals with HH.

A search of the literature identified the same de novo germline mutation in an individual with primary hyper‐aldosteronism and overlapping clinical features which included congenital heart defects (biventricular hypertrophy and ventricular septal defect), seizures and neuromuscular abnormalities. Additional features not present in our patient were pulmonary hypertension, cortical blindness, and primary hyper‐aldosteronism.^7^ The reason(s) for the phenotypic differences between our patient and the patient previously reported with the same mutation are not understood, but may result from genetic and/or environmental modifiers.

In the previously published patient with the same mutation, transient hypoglycaemia of unknown duration was diagnosed on the second day of life and had been treated with diazoxide and hydrocortisone. She had also been born large for gestational age (4.4 kg) consistent with increased insulin secretion in utero.^7^ HH was not reported in a second individual with a different de novo germline *CACNA1D* mutation (p.Ile770Met) in the same series which suggests that the presence of hypoglycaemia may be mutation specific.^7^

Whilst it is possible that the HH in our patient and the previously reported case is unrelated to the *CACNA1D* mutation, the high expression of *CACNA1D* in the pancreas suggests that the mutation is causative of the HH in both individuals.^7^, ^8^ In addition, the presence of HH in 2 of 3 individuals now reported with a germline *CACNA1D* mutation is in keeping with the prevalence of HH in other multisystem diseases such as Timothy syndrome, which results from dominant mutations in *CACNA1C*.^9^

*CACNA1D* encodes an L‐type voltage‐gated calcium channel that has a key role in insulin secretion from pancreatic β‐cells.^8^ Electrophysiological studies have demonstrated that mutant p.Gly403Asp channels activate at lower membrane potentials and exhibit impaired inactivation.^7^ As influx of Ca^2+^ into the β‐cell triggers insulin release following membrane depolarisation, we hypothesize that opening of the channels at a lower membrane potential and a failure to close the channel would result in the dysregulated insulin secretion observed in our patient.

L‐type calcium channels are pharmacologically sensitive to dihydropyridines which bind to and close the channel.^10^ The patient reported by Scholl et al was treated with a dihydropyridine which resulted in a normalization of blood pressure and resolution of the biventricular hypertrophy.^7^ Whilst our patient was successfully treated with diazoxide for 5 years it seems possible that the HH may have also responded to a calcium channel blocker. Treatment with a dihydropyridine might also be effective for the management of the extra‐pancreatic features in our patient.

In conclusion, we report the first mutation detected in a novel disease‐causing gene by NGS in an individual with persistent HH. The identification of an activating p.G403D mutation in 2 unrelated patients with congenital HH supports the role of *CACNA1D* as an aetiological gene for HH, and screening for mutations in this gene should be considered in an individual with HH in addition to heart defects.
